# Tissue expander breast reconstruction outcomes following postmastectomy radiation therapy in the era of neoadjuvant chemotherapy

**DOI:** 10.3389/fonc.2025.1636472

**Published:** 2025-09-02

**Authors:** Oscar Padilla, Meghan Jairam, Amanda Yen, Julie Choi, Elizabeth Buss, Christine Chin, Leah Katz, Jeffery Ascherman, Eileen P. Connolly

**Affiliations:** ^1^ Department of Radiation Oncology, Icahn School of Medicine at Mount Sinai, New York, NY, United States; ^2^ Department of Radiation Oncology, New York-Presbyterian/Columbia University Irving Medical Center, New York, NY, United States; ^3^ Department of Radiology, Brigham and Women’s Hospital, Boston, MA, United States; ^4^ Department of Radiation Oncology, Nuvance Health, Danbury, CT, United States; ^5^ Division of Plastic Surgery, New York-Presbyterian/Columbia University Irving Medical Center, New York, NY, United States

**Keywords:** radiation therapy, breast cancer, oncology, post mastectomy radiation therapy, neoadjuvant chemotherapy, toxicity, complications, tissue expander breast reconstruction

## Abstract

**Background:**

Optimal sequencing of mastectomy, tissue expander breast reconstruction (TE-BR), chemotherapy, and post-mastectomy radiotherapy (PMRT) remains unclear. While PMRT is known to impact TE-BR outcomes, limited data exist comparing outcomes between patients who also receive neoadjuvant chemotherapy (NAC) versus adjuvant chemotherapy (AC).

**Methods:**

A retrospective review of 126 patients diagnosed with invasive breast carcinoma who underwent mastectomy, TE-BR, and PMRT between 2001 and 2017 was conducted. Patients were stratified into NAC (n=74) and AC (n=52) cohorts. Logistic regression and Kaplan-Meier analyses evaluated TE-BR failure rates, completion rates, and radiation toxicity. Multivariate Cox proportional hazard regression modeled TE-BR failure probability.

**Results:**

TE-BR failure rates were significantly higher in the NAC group (44.6% at a median of 18.7 months) compared to the AC group (26.9% at 23.2 months, p=0.041). Moreover, NAC was associated with increased adverse events and lower TE-BR completion rates (both p=0.001). Univariate analysis identified NAC (p=0.007) and acute RT toxicity (p<0.001) as predictors of TE-BR failure. Multivariate analysis confirmed NAC (HR 2.73, p=0.003) and acute RT toxicity (HR 3.16, p<0.001) as independent risk factors. Acute RT toxicity rates were similar between NAC and AC groups (p=0.604). Completing TE expansion before PMRT in NAC patients was linked to higher failure probability (HR 2.58, p=0.023).

**Conclusion:**

Our study is the first to report TE-BR outcomes in women who undergo NAC versus AC in the context of PMRT. Our findings indicate inferior TE-BR outcomes following NAC when PMRT is delivered, emphasizing the importance of shared decision-making between patients and doctors about optimal surgical choice. If eligible, breast conservation or alternate BR technique should be strongly considered in the setting of NAC and future research should explore optimal reconstruction strategies.

## Introduction

The role of post mastectomy radiation therapy (PMRT) and its integration with oncologic breast surgery, as well as timing of reconstruction, and chemotherapy continues to evolve. PMRT improves overall survival and locoregional control in the definitive treatment of breast cancer for select high-risk patients (1–3). PMRT historically is given after completion of adjuvant chemotherapy (AC), at an average of 5–6 months post-mastectomy. When neoadjuvant chemotherapy (NAC) is given however, the treatment window for PMRT following surgery is moved up to optimize oncologic control, often starting as soon as 6–12 weeks post-mastectomy.

The role of NAC has also evolved over the past decade. Initially, NAC offered the benefit of downstaging, allowing breast conservation or rendering inoperable tumors operable. However, now NAC is given to influence a response-guided treatment and even predict ultimate outcome (4, 5). Additionally, the emergence of histologic subtype-guided treatment has influenced the sequencing of chemotherapy with regard to surgery and is now codified in the NCCN guidelines (6). After NAC, timing of breast reconstruction (BR) and PMRT remain an area of continued debate, leading to variable practice patterns that are institutionally driven.

The decision between mastectomy and breast conservation depends on several factors, such as cancer stage and patient preference, socioeconomic status and age (7, 8). Moreover, the number of women undergoing mastectomy for early-stage breast cancer or as a prophylactic intervention in those with genetic predisposition to breast cancer has been increasing in the past decade (9). Due to this, post-mastectomy reconstruction rates have increased, with up to 80% of women pursuing BR, most often opting for tissue expander breast reconstruction (TE-BR) (10, 11).

In the United States, immediate TE-BR represents the most commonly used strategy for oncological BR. Performed as a two-stage procedure, TE-BR consists of immediate placement of a prosthetic expander at the time of surgery, followed by exchange with a permanent silicone or saline implant; either before or after PMRT. National analyses have also shown increasing rates of PMRT in the setting of TE-BR (7).

The aim of this study was to review our patient population from 2001–2017 and identify women who received mastectomy, TE-BR, and who also required PMRT. We report on the aggregate effect of chemotherapy and PMRT on TE-BR outcomes and toxicity. Our median follow-up time from diagnosis of NAC and AC was 4.4 and 5.2 years, respectively. The median follow-up period from TE insertion for NAC and AC was 3.8 and 5.1 years, respectively. Our review occurred during an era of a clinical paradigm shift from primarily AC to NAC for breast cancer management. Here we share our results regarding TE-BR failure rates, completion probability, adverse events, and acute versus chronic PMRT toxicity in these chemotherapy cohorts.

## Methods

A retrospective review was conducted of consecutive breast cancer patients who underwent tissue expander breast reconstruction (TE-BR) following mastectomy and received either neoadjuvant (NAC) or adjuvant (AC) chemotherapy and post-mastectomy radiotherapy (PMRT). The review was conducted in accordance with our Institutional Review Board, approved protocol #AAAJ8512. Each patient was treated by a multidisciplinary breast cancer team, including medical oncology, breast surgical oncology, plastic surgery, and radiation oncology. In this study, we sought to assess the aggregate impact of chemotherapy and PMRT on completion, failure and toxicity rates of TE-BR specifically. As such, patients were excluded if they did not undergo TE-BR, did not complete chemotherapy, or did not complete PMRT. Patients who experienced TE-BR failure and/or opted for an alternate BR approach prior to the start of PMRT were also excluded, as this would not capture the impact of PMRT on TE-BR outcomes in an unconfounded manner.

In our study, the TE-BR period begins with TE insertion at mastectomy and is considered complete on exchange for permanent implant. TE-BR is considered incomplete if permanent implant placement does not occur. TE-BR is defined as a failure if (1) TE is permanently extracted once PMRT has started and BR is aborted or alternate reconstruction technique pursue; (2) or if no additional TE expansions occur within 6 months of completing PMRT, both due to non-oncologic reasons; (3) permanent implant extraction due to non-oncologic reasons; or (4) significant post-implant toxicities including Baker grade IV contraction, severe breast deformity diminishing quality of life documented on serial visits, and recurrent infections/wound healing issues.

CTCAE (Common Terminology Criteria for Adverse Events) guidelines and scoring systems were used to assess toxicity (12). TE-BR adverse events were captured from surgical notes and represent issues that arise prior to permanent implant exchange (i.e., prior to TE-BR completion). TE-BR adverse events include TE leaks, deflations, ruptures, revisions and permanent removals. Acute RT toxicities were captured from radiation oncology notes and represent issues arising during PMRT and up to the first radiation oncology follow-up visit, which is typically 4 weeks status post PMRT completion. Due to the high frequency of low-grade skin issues observed with PMRT, we only reported significant acute RT toxicities such as grade 3 and higher dermatitis, tissue necrosis, infections/cellulitis requiring antibiotics and need for surgical debridement. Chronic post-RT toxicities were captured from radiation oncology notes and represent issues arising after the first radiation oncology follow-up visit. Due to the possibility of multiple adverse events and RT toxicities occurring in the same patient, we tabulated these individually and reported the total number of patients who experienced these events.

TE-BR completion and failure times were calculated from date of TE insertion to date of BR completion or failure respectively, as defined above. The following demographic and clinical variables were assessed: age, cancer stage, body mass index (BMI), race/ethnicity, smoking status, diabetes diagnosis, breast cancer laterality, type of mastectomy, type of lymph node evaluation, RT boost, and regional nodal irradiation (RNI). Comparisons of these variables between NAC and AC cohorts were performed using the Chi-squared test. A logistic regression model with odds ratio (OR) was used to analyze the relationship of chemotherapy and radiotherapy cohorts with TE-BR outcomes and RT toxicities. The Kaplan-Meier method was used to calculate the probability of tumor control and survival along with hazard ratios (HR) and 95% confidence intervals (CI). Probability of free-of-BR-failure (FBRF) curves were compared in univariate analysis using the log-rank test. Only variables that were found to be significant with p -value less than 0.05 on univariate analyses were considered in multivariate analysis. Multivariate analysis was performed by Cox proportional hazards regression. Differences in time-to-event intervals between NAC and AC cohorts were analyzed by the Mann-Whitney test.

## Results

### Patient characteristics

A total of 126 patients with biopsy-proven breast cancer underwent mastectomy with TE-BR and received chemotherapy and PMRT (**Table 1**). Seventy-four of these patients received NAC, while 52 received AC. Median follow-up time from diagnosis was 4.4 and 5.2 years for NAC and AC cohorts, respectively. There was no difference in mastectomy type between NAC and AC cohorts (p=0.87; **Table 1**). Modified radical and total mastectomies decreased over time while nipple- and skin-sparing mastectomies increased for both cohorts (**Supplementary Figure 1**). Mastectomy was accompanied by sentinel lymph node biopsy (SLNB) in 15.9% of patients and by axillary lymph node dissection (ALND) in 84.1% of patients (**Table 1**). Subpectoral tissue expander placement occurred in all but 1 patient. All patients received adjuvant PMRT to the affected chest wall, with a median and modal dose of 5000 cGy (range 4005–5040 cGy). Approximately 95% of patients also underwent RNI and 45.2% received a focal boost, with a median dose of 1000 cGy. There was no significant difference with respect to SLNB/ALND, RNI, RT boost, cancer stage, median age, race, diabetes diagnosis or obesity status between NAC and AC cohorts (**Table 1**). Eight of the 74 NAC patients were current smokers versus none in the AC cohort (p=0.02; **Table 1**).

**Table 1:** Demographics according to chemotherapy cohort.

Covariates	Total	Chemotherapy		
		NAC	AC	pX²
	126 (100%)	74 (58.7%)	52 (41.3%)	
Age (median)				
<= 48 years	66 (52.4%)	35	31	0.18
>48 years	60 (47.6%)	39	21	
Stage				
I-II	62 (49.2%)	35	27	0.61
III-IV	64 (50.8%)	39	25	
BMI				
<30	70 (55.6%)	40	30	0.69
>=30	56 (44.4%)	34	22	
Race				
White	45 (35.7%)	27	18	0.83
non-White	81 (64.3%)	47	34	
Smoking				
Not current	118 (93.7%)	66	52	0.02
Current	8 (6.3%)	8	0	
Diabetes				
No	106 (84.1%)	64	42	0.39
Yes	20 (15.9%)	10	10	
Laterality				
Right	61 (48.4%)	35	26	0.78
Left	65 (51.6%)	39	26	
Mastectomy Type				
MR/TM	52 (41.3%)	31	21	0.87
SS/NS	74 (58.7%)	43	31	
Lymph Node Dissection				
SLNB	20 (15.9%)	12	8	0.90
ALND	106 (84.1%)	62	44	
RT boost				
No	69 (54.8%)	36	33	0.10
Yes	57 (45.2%)	38	19	
Regional Nodal RT				
No	6 (4.8%)	5	1	0.21
Yes	120 (95.2%)	69	51	

NAC, neoadjuvant chemotherapy; AC, adjuvant chemotherapy; Chi-square (*X^2^*) analysis performed with reported *p* value; MR, modified radical mastectomy; TM, total mastectomy; SS, skin-sparing mastectomy; NS, nipple-sparing mastectomy; SNLB, sentinel lymph node biopsy; ALND, axillary lymph node dissection.

### Breast reconstruction outcomes

Approximately 37% of all patients experienced TE-BR failure at a median of 20.1 months following TE insertion (**Table 2**). NAC patients experienced a BR failure rate of 44.6% at a median of 18.7 months (range 4.57 - 76.5) following TE insertion versus 26.9% at a median of 23.2 months (range 12.7 - 116.2) for AC patients (p=0.041; **Table 2**). Primary reasons for TE-BR failure in each cohort as listed in **Supplementary Table S1**. Moreover, 18.9% of NAC patients failed to complete BR, compared to 1.9% of AC patients (p=0.001; **Table 2**). BR adverse events occurred in 23% and 3.8% of NAC and AC patients respectively (p=0.001; **Table 2**). Specific TE-BR adverse events are listed in **Supplementary Table S2**. Receipt of NAC (**Table 2**) was found to be significantly associated with BR failure (OR 2.19, 95% CI 1.02 - 4.70), lower BR completion status (OR 0.08, 95% CI 0.01 - 0.66) and BR adverse events (OR 7.46, 95% CI 1.64 - 33.9). Acute RT toxicities (**Table 2**) occurred in 28.4% of NAC patients and in 32.7% of AC patients, but this difference was not statistically significant (p=0.643; **Table 2**). Rates of chronic post-RT toxicities (**Table 2**), on the other hand, were significantly different between NAC and AC cohorts, at 90.5% and 65.4% respectively (p=0.001; **Table 2**). Receipt of RT boost was not associated with BR outcomes or RT toxicities (**Supplementary Table S3**).

**Table 2:** Breast reconstruction (BR) and RT outcomes according to chemotherapy cohort.

Outcome	Total	NAC cohort	AC cohort	Odds Ratio (95% CI)	*p-value*
	126 (100%)	74 (58.7%)	52 (41.3%)		
BR Failure					
No	79 (62.7%)	41 (55.4%)	38 (73.1%)	2.19 (1.02-4.70)	0.041
Yes	47 (37.3%)	33 (44.6%)	14 (26.9%)		
*Median time to failure*	*20.1 months*	*18.7 months*	*23.2 months*		
BR Completion					
No	15 (11.9%)	14 (18.9%)	1 (1.9%)	0.08 (0.01-0.66)	0.001
Yes	111 (88.1%)	60 (81.1%)	51 (98.1%)		
*Median time to completion*	*15.3 months*	*16.1 months*	*14.8 months*		
BR Adverse Event					
No	107 (84.9%)	57 (77.0%)	50 (96.2%)	7.46 (1.64-33.9)	0.001
Yes	19 (15.1%)	17 (23.0%)	2 (3.8%)		
Acute RT Toxicity					
No	88 (69.8%)	53 (71.6%)	35 (67.3%)	0.82 (0.38-1.76)	0.604
Yes	38 (30.2%)	21 (28.4%)	17 (32.7%)		
Chronic RT Toxicity					
No	25 (19.8%)	7 (9.5%)	18 (34.6%)	5.07 (1.93-13.3)	0.001
Yes	101 (80.2%)	67 (90.5%)	34 (65.4%)		

NAC, neoadjuvant chemotherapy; AC, adjuvant chemotherapy; Logistic regression performed for odds ratio with NAC as reference group; CI, confidence interval.

### Univariate and multivariate analyses of BR failure

**Figure 1:**
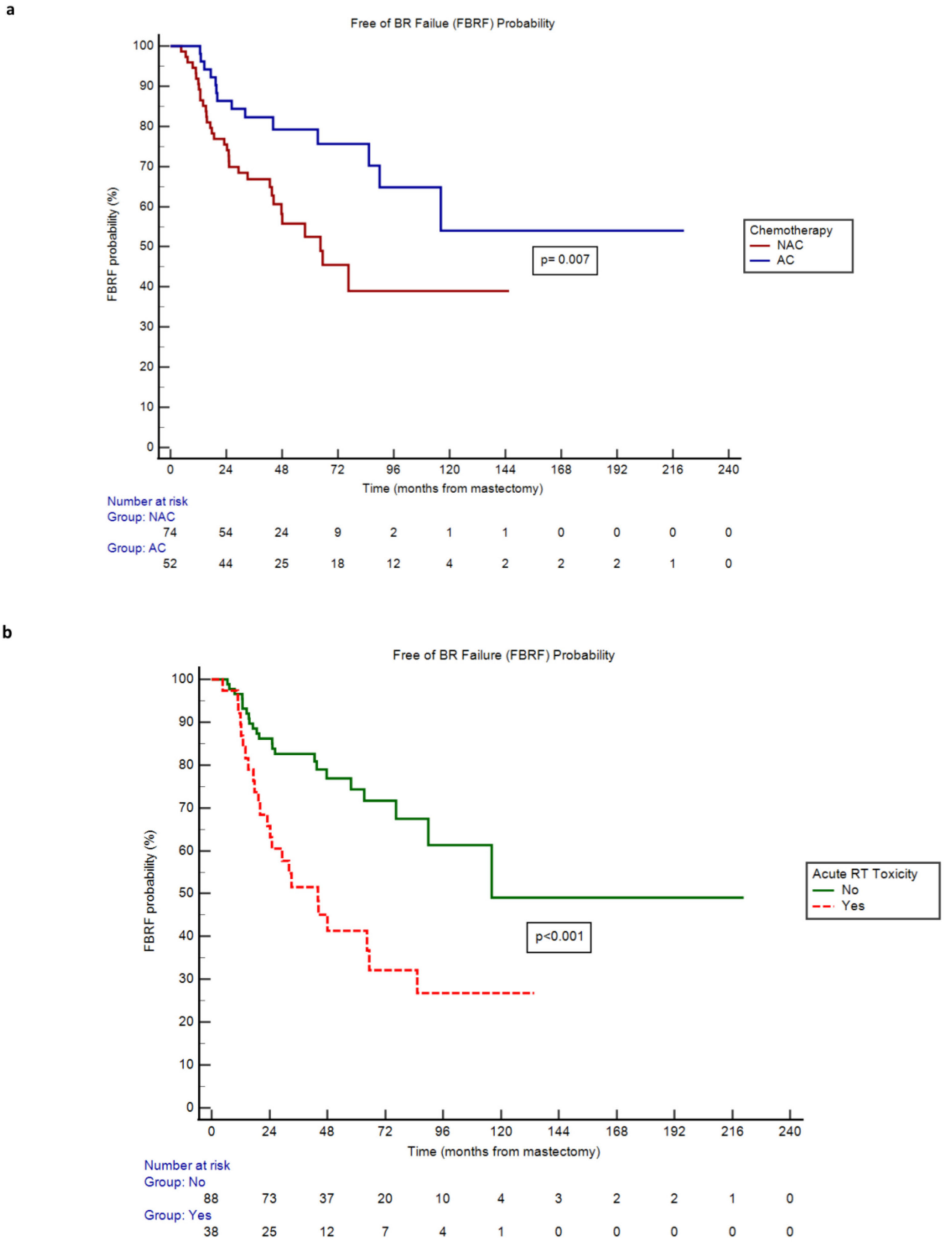
**(a)** Free of BR Failure (FBRF) Probability of neoadjuvant (NAC) versus adjuvant (AC) chemotherapy cohorts over time. **(b)** Free of BR Failure (FBRF) Probability of patients with versus without acute radiation (RT) toxicity over time.

By Kaplan-Meier method, receipt of NAC was associated with a higher hazard rate (HR) of BR failure versus receipt of AC (HR 2.24, 95% CI 1.25 - 4.03, p=0.007; **Table 3**). In line with this, median Free of Breast Reconstruction Failure (FBRF) probability was 64.5 months in the NAC cohort and not yet to be reached in the AC cohort (**Figure 1a**). Moreover, patients who sustained acute RT toxicities experienced a higher risk of BR failure and lower FBRF probability versus those who did not (HR 3.39, 95% CI 1.78 - 6.47, p<0.001; **Table 3**, **Figure 1b**). A similar correlation was observed for patients who developed BR adverse events versus those who did not (HR 6.79, 95% CI 2.63 - 17.51, p ≤ 0.001; **Table 3**). While the remainder of covariates explored were not significantly associated with BR failure, there was a trend toward higher BR failure risk in obese (BMI >/= 30) versus non-obese patients (p=0.051; **Table 3**). Finally, for NAC patients who completed final TE expansion prior to the start of RT, there was a higher BR failure risk versus NAC patients who completed final expansion after RT (HR 2.58, 95% CI 1.14 - 5.84, p=0.023; **Table 3**, **Supplementary Figure 2**); this association was not observed in the AC cohort.

**Table 3:** Univariate analysis for breast reconstruction (BR) failure.

Covariates	HR (95% CI)	p-value
acute RT toxicity: yes vs no	HR 3.39 (1.78-6.47)	<0.001
BR adverse event: yes vs no	HR 6.79 (2.63-17.51)	<0.001
Chemotherapy: NAC vs AC	HR 2.24 (1.25-4.03)	0.007
Body Mass Index: ≥30 vs <30	HR 1.79 (1.00-3.20)	0.051
Regional nodal RT: yes vs no	HR 1.86 (0.51-6.79)	0.345
Lymph node dissection: SLNB vs ALND	HR 1.13 (0.51-2.51)	0.758
smoking (current): yes vs no	HR 1.53 (0.39-6.09)	0.544
age median: <=48 vs >48 yrs	HR 0.87 (0.49-1.54)	0.628
RT boost: yes vs no	HR 0.79 (0.45-1.41)	0.426
laterality: left vs right	HR 1.16 (0.65-2.05)	0.620
stage: I-II vs >II	HR 1.11 (0.63-1.97)	0.717
mastectomy type: MR/TM vs SS/NS	HR 0.76 (0.43-1.37)	0.364
race: white vs nonwhite	HR 0.93 (0.51-1.71)	0.823
diabetes: yes vs no	HR 1.08 (0.51-2.27)	0.842
final expansion RT relation: before or after RT	HR 1.06 (0.59-1.88)	0.857
[NAC subset] final expansion RT relation: before or after RT	HR 2.58 (1.14-5.84)	0.023
[AC subset] final expansion RT relation: before or after RT	HR 0.76 (0.26-2.27)	0.624

NAC, neoadjuvant chemotherapy; AC, adjuvant chemotherapy; MR, modified radical mastectomy; TM, total mastectomy; SS, skin-sparing mastectomy; NS, nipple-sparing mastectomy; SNLB, sentinel lymph node biopsy; ALND, axillary lymph node dissection; Kaplan-Meier performed for hazard ratio (HR); CI, confidence interval.

Covariates significant on univariate analysis were entered in a multivariate Cox model. The ‘BR adverse event’ covariate was excluded to prevent confounding by collinearity, as prior logistic regression analysis had identified a strong correlation with the type of chemotherapy received. Multivariate analysis showed that NAC and acute RT toxicity remained significantly associated with risk of BR failure (p ≤ 0.001; **Supplementary Table S4**).

### Time interval analyses

The median interval from mastectomy to start of RT was 2.07 months (range 1.90 - 2.37 months) for NAC patients versus 7.47 months (range 7.17 - 7.82 months) for AC patients (p ≤ 0.001; **Table 4**). For NAC patients who sustained pathologic complete response, median interval from mastectomy to RT start was longer than for NAC patients who had partial or no response to chemotherapy: median 94 versus 59.5 days (p=0.010; **Supplementary Table S5**). There was no significant difference in mastectomy to first TE expansion intervals between NAC and AC cohorts (p=0.499). However, NAC patients had a shorter interval between first TE expansion and the start of RT when compared to AC patients: median 1.88 versus 6.32 months respectively (p ≤ 0.001; **Table 4**). For NAC patients who completed their final TE expansion before the start of RT (28.4%), they had a shorter interval between these timepoints than AC patients who completed their final TE expansion before the start of RT (65.4%): median 0.70 versus 3.72 months respectively (p ≤ 0.001; **Table 4**). Conversely, for NAC patients who completed their final TE expansion after the end of RT (71.6%), they had a longer interval between these timepoints than AC patients who completed their final TE expansion after the end of RT (34.6%): median 5.57 versus 3.52 months respectively (p=0.018; **Table 4**). Collectively, there was no significant difference in total TE expansion time between NAC and AC cohorts: median 4.55 versus 5.15 months respectively (p=0.278; **Table 4**). Similarly, there was no significant difference in oncologic package time (OPT), which represents the sum of chemotherapy, mastectomy and RT durations, between NAC and AC cohorts: median 9.00 versus 8.65 months (p=0.615; **Table 4**). However, total treatment time, which represents OPT plus time to permanent implant placement from mastectomy, did differ significantly between NAC and AC patients who completed BR: median 18.2 versus 13.9 months respectively (p ≤ 0.001; **Table 4**).

**Table 4:** Time intervals of interest.

TIME INTERVALS	NAC cohort	AC cohort	Mann-Whitney p-value
	Median in months (95% CI)		
Mastectomy to RT start	2.07 (1.90-2.37)	7.47 (7.17-7.82)	<0.001
Mastectomy to 1st TE expansion	1.10 (0.93-1.23)	1.03 (0.93-1.23)	0.499
1st TE expansion to RT start	1.88 (1.34-2.30)	6.32 (5.73-6.54)	<0.001
Final TE expansion to RT start *(when final expansion occurs pre-RT)*	0.70 (0.45-1.16) *(n=21/74)*	3.72 (2.14-4.53) *(n=34/52)*	<0.001
RT end to final TE expansion *(when final expansion occurs post-RT)*	5.57 (3.89-6.43) *(n=53/74)*	3.52 (2.20-5.14) *(n=18/52)*	0.018
Total TE expansion time	4.55 (2.92-5.99)	5.15 (3.85-6.27)	0.278
Oncologic package time (OPT): *chemo + mastectomy + RT duration*	9.00 (8.46-9.36)	8.65 (8.46-9.09)	0.615
Total treatment time: *OPT + time to implant placement*	18.2 (17.5-20.0) *(n=60/74)*	13.9 (13.3-16.5) *(n=51/52)*	<0.001

NAC, neoadjuvant chemotherapy; AC, adjuvant chemotherapy; CI, confidence interval.

## Discussion

Currently, no level I evidence definitively guides the optimal sequencing of breast reconstruction (BR) and postmastectomy radiation therapy (PMRT) in the context of neoadjuvant chemotherapy (NAC) versus adjuvant chemotherapy (AC). Chemotherapy itself has been reported to influence postoperative complications by altering vascular remodeling, delaying wound healing, and increasing susceptibility to infections (13–15). These complications are further exacerbated by the well-documented morbidity of PMRT in women undergoing tissue expander (TE) and implant-based BR (16, 17). Previous studies have demonstrated the compounded adverse effects of chemotherapy and PMRT on BR outcomes. For example, Lam et al. reported that both chemotherapy and PMRT independently increased the risk of TE or implant loss, with their combination further elevating the probability of failure (18). Pathophysiologically, the sequence of chemotherapy followed by PMRT leads to reduced type I collagen deposition, compromised extracellular matrix formation, fibrous encapsulation, and impaired neovascularization, all of which can contribute to poor BR outcomes (19).

The increasing use of NAC has prompted questions about its influence on TE-BR success rates and complications, especially in patients who require PMRT. Prior studies suggest that NAC recipients are less likely to undergo BR with TE or permanent implants, even after adjusting for age and disease stage, raising concerns about inherent morbidity associated with NAC (20). Given these findings, it is therefore imperative to explore the impact of NAC in conjunction with PMRT on TE-BR, the most popular of reconstruction techniques (10). However, existing literature is scarce on this topic. Available literature is difficult to interpret due to heterogeneous chemotherapy regimens, evolving systemic agents, and varying BR techniques (immediate versus delayed, prosthetic versus autologous). Moreover, inconsistent definitions of BR failure across studies make it difficult to draw meaningful clinical conclusions or recommendations for patients who undergo chemotherapy and PMRT.

To our knowledge, this study is the first to directly compare TE-BR outcomes in NAC versus AC patients requiring PMRT. Our findings indicate that NAC is associated with higher BR failure rates, delayed or unsuccessful BR completion, and increased complications. We report a TE-BR failure rate of 44.6% in the NAC cohort compared to 26.9% in the AC cohort. Our study uses a patient-centric definition of BR failure and consists of not only permanent implant extraction but endpoints of lack of BR success that reflect diminished quality-of-life (e.g., grade 3–4 toxicities). Prior studies have reported variable TE-BR failure rates, ranging from 32 - 40% in NAC patients (21–23) and on the order of 20% in AC patients (24, 25) receiving PMRT. Like our findings, these studies demonstrate a generally higher incidence of TE-BR failure following NAC.

Additional key findings of our study include a nearly 20% lower BR completion rate as well as 20% higher adverse events in women who receive NAC versus AC followed by PMRT. Findings in the literature regarding the relative impact of NAC versus AC on BR outcomes and morbidity are inconsistent. Peled et al., for instance, reported no significant differences in immediate BR complications requiring unplanned reoperations (approximately 30%) or in TE/implant loss rates (approximately 20%) between NAC and AC groups (11). In contrast, a study by Dolen et al. found higher rates of premature TE removal in AC patients (19.9%) as compared to NAC (17.3%) and no-chemotherapy cohorts (12.5%) (24). While meta-analyses on this topic are limited, one such study by Song et al., concluded that NAC was not associated with increased BR complications. However, this meta-analysis notably did not perform subgroup stratification within the control arm, which consisted of AC and no-chemotherapy patients alike, thus limiting a direct comparison between NAC and AC morbidity (26). Moreover, the lack of stratification by receipt of PMRT, or not, in these prior studies represents another major shortcoming, which severely limits direct comparisons to our findings.

Regarding reconstruction completion, a study by Lin et al., reported an 80% TE-BR completion rate in PMRT recipients, which is comparable to our overall 88% completion rate (27). Additionally, Cordeiro et al. found that 90% of AC patients in their study achieved TE-BR completion in the setting of PMRT, like our AC cohort’s 98% rate (28). Considering the relatively recent incorporation of NAC into the reconstruction-PMRT regime, there is a dearth of studies specifically reporting on BR completion rates in this chemotherapy cohort. However, prior studies do report a lower likelihood of pursuing immediate prosthetic BR following NAC and a slight preference for delayed autologous BR in this cohort, citing treatment fatigue as a likely explanation for these observations (20, 24). Surprisingly, in our study, we found no significant difference in oncologic package time between chemotherapy cohorts, suggesting that cancer treatment duration likely does not play a major role in possible fatigue and lack of BR completion. However, we did observe that the time from mastectomy to BR completion was significantly longer for women in the NAC group compared to the AC group (18.2 months vs. 13.9 months), largely due to a significantly protracted interval between the end of PMRT and implant exchange.

The timing of tissue expansion and implant exchange are critical factors, particularly for NAC patients who require PMRT. In the AC setting, tissue expansions occur gradually over 5–6 months while patients undergo chemotherapy, allowing the TE to reach full expansion before PMRT. Implant exchange then typically takes place about 6 months after radiation. In contrast, for NAC patients, the push to achieve local tumor control drives PMRT to start within 12 weeks post-mastectomy, significantly compressing the timeline for tissue expansion. As a result, in our study, NAC patients had a significantly shorter interval between first tissue expansion and the start of PMRT at 1.88 months, compared to 6.32 months in AC patients. This accelerated expansion timeline may have contributed to increased tissue stress and a higher risk of BR adverse events and failure. This hypothesis is supported by the higher hazard of failure observed in the subset of NAC patients who completed tissue expansion and subsequent implant exchange speedily prior to the start of PMRT. Our findings indicate that the shorter mastectomy-to-PMRT interval inherent in the NAC regimen (median 2.07 months vs. 7.47 months in AC) may play a role in these complications. In contrast, the longer mastectomy-to-PMRT interval in AC patients allows for more gradual tissue expansion, potentially mitigating some of these risks.

Our study also highlights the impact of radiation-related toxicity on TE-BR failure. Standard CTCAE (Common Terminology Criteria for Adverse Events) guidelines and scoring systems were used to assess toxicity. Acute radiation toxicity was found to be a significant predictor of BR failure (HR 3.39, p<0.001), which highlights the importance of following these patients closely and managing side-effects effectively as they occur during PMRT. While previous studies, such as Naoum et al., have reported chest wall boosts as independent predictors of implant failure (23), we found no significant association between radiation boost and BR outcomes. A potential explanation may be the use of skin bolus in the majority of patients who received a boost in the prior study; in the present study, we did not collect or analyze this variable since the addition of a boost would be at the discretion of the treating physician based on risk factors and independent of the timing of chemotherapy. Lastly, while acute radiation toxicity did not differ between NAC and AC cohorts in our study, chronic toxicity was significantly increased in the former, prompting the need for long-term surveillance and referral to physical therapy, surgery and/or ancillary survivorship services in this group.

Given the increased complications observed with TE-BR in the NAC-PMRT setting, alternative reconstruction techniques may be preferable. The choice of reconstruction method significantly influences complication risk. A meta-analysis by Barry et al. found that TE-BR in PMRT recipients carried a fivefold higher risk of complications compared to autologous reconstruction (29). Moreover, a recent systematic review and meta-analysis of immediate vs delayed autologous breast reconstruction reported comparable complication rates in the setting of PMRT. This suggests that immediate reconstruction with an autologous flap, despite concurrent PMRT, may offer patient-centered benefits, such as reducing the emotional and physical burden of multiple surgeries by consolidating procedures into a single operation (30). Additional studies have corroborated the higher morbidity associated with TE-BR compared to autologous approaches, particularly in NAC patients (23, 31, 32). Other studies have demonstrated that patients undergoing direct-to-implant (DTI) reconstruction experience lower overall complication rates than staged TE/implant reconstructed patients, regardless of radiation history. The single-stage DTI approach eliminates the need for a second surgery and is often regarded as a more economical and practical method for immediate implant-based reconstruction (33). Naoum et al. also reported superior outcomes with DTI compared to TE-BR, with failure rates of 10% and 40%, respectively (23).

However, alternative BR techniques such as autologous reconstruction carry their own risks, including flap necrosis and revision surgery rates of 10-30% in PMRT recipients (31, 34–36). One strategy to mitigate these complications is to sequence radiation therapy before mastectomy and autologous reconstruction—an approach that has shown promise in previous studies (37). Another emerging option is autologous fat grafting (AFG), which has been shown to have regenerative potential attributed to its adipose-derived stem cells (38). AFG has been shown to promote angiogenesis, peripheral nerve regeneration, enhance dermal thickness and elasticity and has also shown promise in reversing radiation-associated dermal fibrosis (39, 40). While a comprehensive review of all surgical options is beyond the scope of this study, to our knowledge, no current data are sufficient to definitively favor one reconstructive method over another.

In short, our study demonstrates that in the setting of PMRT, NAC is associated with increased BR failure, delayed or incomplete BR completion, and higher rates of adverse events. The inclusion of patients treated over a period of 16 years strengthens our results by encompassing a broad spectrum of chemotherapy regimens and surgical techniques. Notably, despite advances in surgical methods during the NAC era, TE-BR outcomes remained inferior in NAC patients, reinforcing our hypothesis that the condensed mastectomy-to-PMRT interval as a result of NAC increases BR failure risk.

Limitations of this study include its retrospective single-institution design and variability in surgical techniques over time. Moreover, hypofractionated PMRT was not used during our study period, and how this schedule affects TE-BR outcomes after chemotherapy remains an important question to be answered, particularly as emerging data suggests reconstruction isotoxicity versus patients undergoing conventional PMRT (41). In addition, our cohort was very defined and only included TE-BR patients who completed both chemotherapy and PMRT, in order to assess the compounded impact of these oncologic therapies on reconstruction outcomes. Along this same line, patients who failed TE-BR prior to PMRT were excluded as this would confound the latter assessment. Lastly, our definition of BR failure, which includes lack of BR completion as well as endpoints beyond permanent implant extraction (e.g., severe toxicity), may be more conservative than other studies leading to comparatively slightly higher rates of BR failure. This definition of BR failure was selected given its real-world applicability with regard to patient-centered outcomes and healthcare resources.

As breast cancer treatment paradigms evolve, the interplay between PMRT, BR techniques, and patient quality of life remains paramount. Patients undergoing NAC should be counseled on the risks of TE-BR and PMRT, and discussions should include alternative reconstruction options, including autologous BR or breast conservation when eligible. Future prospective studies are warranted to validate our findings and establish the optimal reconstruction technique and integration schema with oncologic therapies in this population.

## Data Availability

The raw data supporting the conclusions of this article will be made available by the authors, without undue reservation.
