# Supplementary material for: Tissue expander breast reconstruction outcomes following postmastectomy radiation therapy in the era of neoadjuvant chemotherapy
Source: Front Oncol. 2025 Sep 2;15:1636472. doi: 10.3389/fonc.2025.1636472 (PMC12436492; doi:10.3389/fonc.2025.1636472)
Supplement: Supplementary file 1 [file DataSheet1.docx]

**Supplementary Table 1.** TE-BR primary failure reasons for NAC and AC cohorts.

| **TE-BR Failure Primary Reasons** | | | | | | | |
| --- | --- | --- | --- | --- | --- | --- | --- |
| **Chemotherapy Cohort** | **Total patients** | **recurrent infection/ wound healing issue** | **severe breast deformity** | **contracture (grade 4)** | **permanent TE removal** | **no TE-implant exchange within 6 months of PMRT** | **implant deflation/rupture** |
| NAC (n=74) | 33 (44.6%) | 1 | 8 | 5 | 13 | 4 | 2 |
| AC (n=52) | 14 (26.9%) | 1 | 9 | 2 | 0 | 1 | 1 |

**Supplementary Table 2.** Summary of TE-BR adverse events for NAC and AC cohorts.

| **TE-BR Adverse Events  (pre-implant exchange)** | | | | | | | | |
| --- | --- | --- | --- | --- | --- | --- | --- | --- |
| **Chemotherapy Cohort** | **Total patients** | **TE leak** | **TE deflation** | **TE rupture** | **infection/ wound healing issue** | **chest tightness requiring TE surgical revision** | **breast deformity requiring TE surgical revision** | **requiring permanent TE removal** |
| NAC (n=74) | 17 (23%) | 1 | 1 | 1 | 6 | 5 | 3 | 13 |
| AC (n=52) | 2 (3.8%) | 1 | 1 | 0 | 0 | 0 | 1 | 0 |

**Supplementary Table 3.** Breast reconstruction (BR) and RT outcomes according to RT boost. NAC= neoadjuvant chemotherapy. AC= adjuvant chemotherapy. Logistic regression performed for odds ratio with NAC as reference group. CI = confidence interval.

| **Covariates** |  | **RT Boost** | |  |  |  |
| --- | --- | --- | --- | --- | --- | --- |
|  | **Total** | **Yes** | **No** | **Odds Ratio (95% CI)** | **p-value** |  |
| **BR Failure** |  |  |  |  |  |  |
| **Combined** | 126 (100%) | 57 (45.2%) | 69 (54.8%) |  |  |  |
| **No** | 79 (62.7%) | 37 (64.9%) | 42 (60.9%) | 0.84 (0.41-1.74) | 0.640 |  |
|  |  |  |  |  |  |  |
| **Yes** | 47 (37.3%) | 20 (35.1%) | 27 (39.1%) |  |  |  |
|  |  |  |  |  |  |  |
| **BR Completion** |  |  |  |  |  |  |
| **No** | 15 (11.9%) | 5 (8.8%) | 10 (14.5%) | 1.76 (0.57-5.49) | 0.318 |  |
|  |  |  |  |  |  |  |
| **Yes** | 111 (88.1%) | 52 (91.2%) | 59 (85.5%) |  |  |  |
|  |  |  |  |  |  |  |
| **BR Adverse Event** |  |  |  |  |  |  |
| **No** | 107 (84.9%) | 51 (89.5%) | 56 (81.2%) | 0.51 (0.18-1.43) | 0.188 |  |
|  |  |  |  |  |  |  |
| **Yes** | 19 (15.1%) | 6 (10.5%) | 13 (18.8%) |  |  |  |
|  |  |  |  |  |  |  |
| **Acute RT Toxicity** |  |  |  |  |  |  |
| **No** | 88 (69.8%) | 41 (71.9%) | 47 (68.1%) | 0.83 (0.39-1.80) | 0.643 |  |
|  |  |  |  |  |  |  |
| **Yes** | 38 (30.2%) | 16 (28.1%) | 22 (31.9%) |  |  |  |
|  |  |  |  |  |  |  |
| **Chronic RT Toxicity** |  |  |  |  |  |  |
| **No** | 25 (19.8%) | 13 (22.8%) | 12 (17.4%) | 0.71 (0.30-1.71) | 0.499 |  |
|  |  |  |  |  |  |  |
| **Yes** | 101 (80.2%) | 44 (77.2%) | 57 (82.6%) |  |  |  |
|  |  |  |  |  |  |  |

**
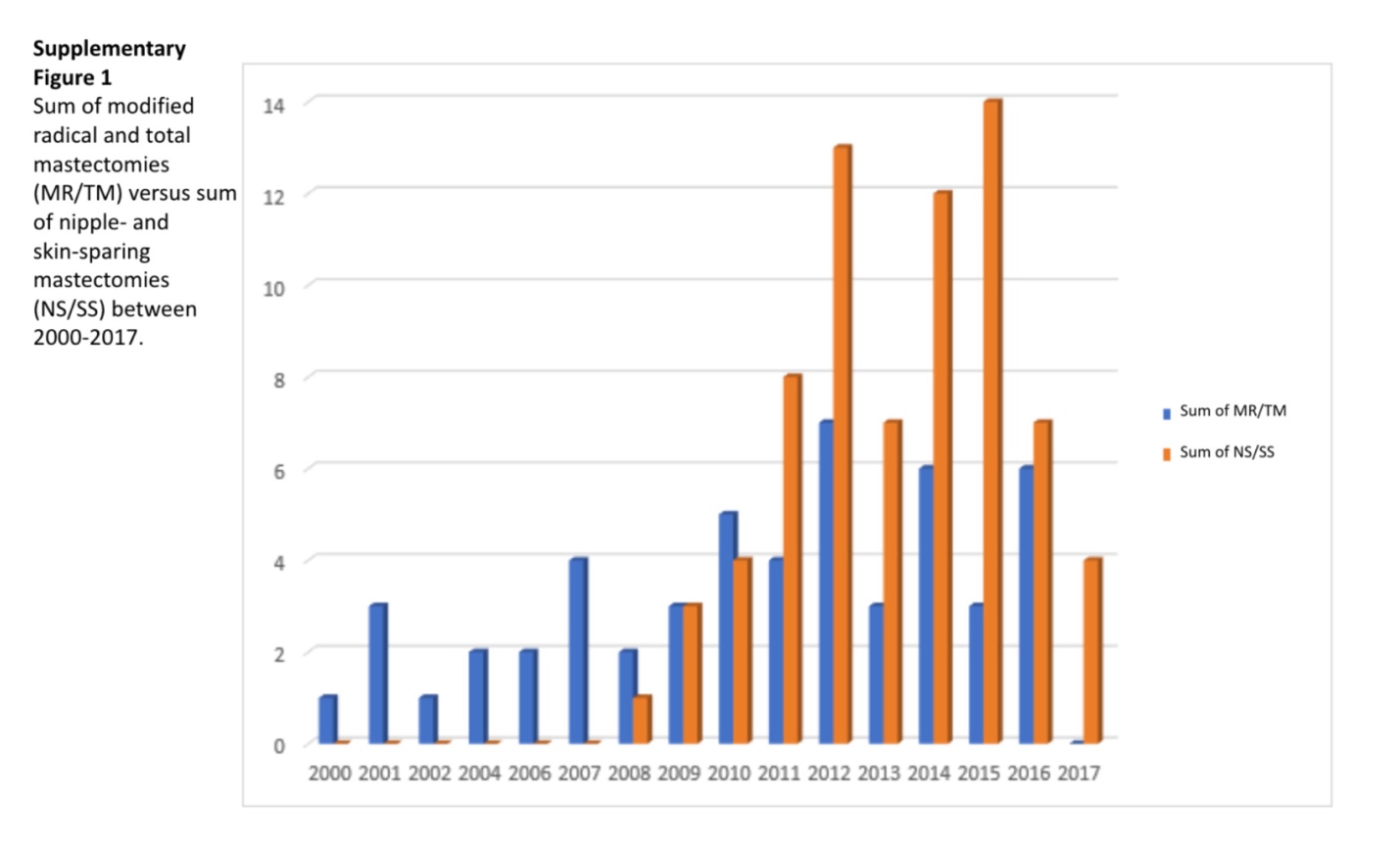
**

**
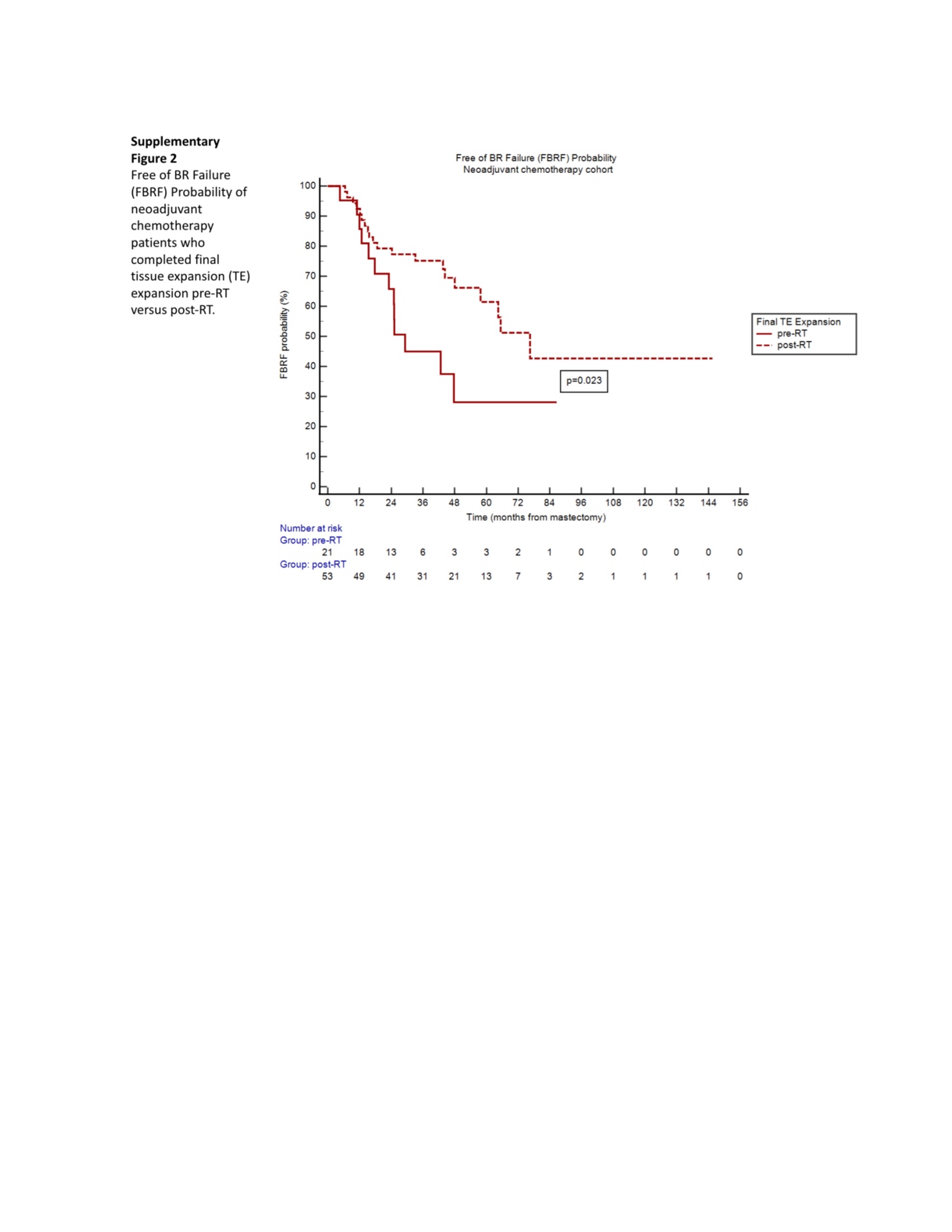
**

**Supplementary Table 4.** Multivariate analysis for breast reconstruction (BR) failure. NAC= neoadjuvant chemotherapy. AC= adjuvant chemotherapy. Cox-proportion model performed for hazard ratio (HR). CI = confidence interval.

| **Covariates** | **HR (95% CI)** | **p-value** | **Overall model  p-value** |
| --- | --- | --- | --- |
| acute RT toxicity: yes vs no | 3.16  (1.77-5.63) | <0.001 | <0.001 |
| Chemotherapy:  NAC vs AC | 2.73  (1.42-5.27) | 0.003 |  |

| **NAC Cohort  (n=74)** | **Patients (%)** | **Median Time Mastectomy to RT Start (95% CI)** | **Mann-Whitney p-value** |
| --- | --- | --- | --- |
| **Pathologic Complete Responses (pCR)** | 10  (13.5%) | 94 days (58.3-122.8) | 0.010 |
| **Pathologic Partial/No Response** | 64  (86.5%) | 59.5 days (55.7-65.2) |  |

**Supplementary Table 5.** Median interval from mastectomy to start of RT for neoadjuvant chemotherapy (NAC) cohort. CI = confidence interval.
